# Platelet-rich plasma and folliculogenesis: a systematic mechanistic review and computational pathway analysis

**DOI:** 10.3389/frph.2026.1883067

**Published:** 2026-07-30

**Authors:** Adamantia Kontogeorgi, George-Konstantinos Papaioannou, Fanourios Makrygiannakis, Ioannis Boutas, Antonis Makrigiannakis, Sophia N. Kalantaridou

**Affiliations:** 1 IVF Serum, Advanced IVF Treatment Center, Likovrisi, Athens, Greece; 2 Department of Obstetrics and Gynecology, Medical School, University of Crete, Heraklion, Greece; 3 Department of Obstetrics and Gynecology, Medical School, National and Kapodistrian University of Athens, Athens, Greece

**Keywords:** folliculogenesis, PI3K–AKT pathway, platelet-rich plasma (PRP), protein–protein interaction network analysis, receptor tyrosine kinase signaling

## Abstract

**Introduction:**

Platelet-rich plasma (PRP) has been proposed as a potential intervention for promoting ovarian follicular development. However, its underlying biological mechanisms remain unclear. This study investigated whether the mechanisms through which PRP may influence follicular development are consistent with previously established regulatory mechanisms of human folliculogenesis.

**Methods:**

A systems biology approach was used to examine the mechanistic relationships between growth factors present in PRP and known regulators of human follicular development. Gene databases were compared, and pathway enrichment analysis and integrated network modeling were performed to identify shared signaling pathways and patterns of molecular connectivity.

**Results:**

Although there was limited overlap between the two gene databases, substantial convergence was observed in pathway enrichment and integrated network modeling. This convergence involved receptor tyrosine kinase-centered intracellular signaling pathways, particularly the PI3K–AKT and MAPK pathways. Network topology demonstrated coherent connectivity between extracellular growth factors and established survival and proliferation signaling axes. However, no overlap was identified between genes associated with PRP and genes encoding oocyte-specific transcriptional regulators involved in primordial follicle formation and differentiation.

**Discussion:**

These findings support a model in which PRP does not replace the endogenous transcriptional program governing follicular development. Instead, PRP may increase upstream growth factor signaling and enhance intrinsic survival and proliferation pathways within the ovarian microenvironment. This systems-level framework provides mechanistic insight into the biological plausibility of PRP use in reproductive medicine and offers a rational basis for targeted experimental validation.

## 1 Introduction

Folliculogenesis is a dynamic and complex biological process during which ovarian follicles develop from the primordial stage to full maturation and ovulation. This process is fundamental to female fertility, since it determines the number and quality of oocytes available for fertilization (1). The regulation of folliculogenesis depends on an advanced network of endocrine, paracrine, and autocrine signals that coordinate interactions among the oocyte, ovarian somatic cells, and the surrounding stromal and vascular microenvironment (2–4). Oocyte and granulosa cell development occurs concurrently and is highly interdependent, rendering communication between these cell populations essential for proper follicle maturation. Seminal studies by Eppig et al. demonstrated that mammalian oocyte development depends on continuous bidirectional communication with surrounding somatic cells, supporting the concept of the follicle as a functional unit rather than a collection of independent cell types (5–7). This communication is mediated by direct cell contacts, such as gap junctions, and by paracrine signals from the oocyte, including GDF9 and BMP15 (3). The epidermal growth factor receptor (EGFR) axis also plays a central role in regulating granulosa–oocyte communication (8, 9) by integrating signals from EGF-like ligands and promoting the functional maturation of the oocyte–granulosa cell complex (10).

In addition to gonadotropins and oocyte-related factors, growth factors such as PDGF, FGF, IGF, TGF-β, and VEGF have significant roles in controlling follicular development (11). Growth factor activity affects cell growth, viability, differentiation, blood vessel formation, and the structure and/or content of extracellular matrix remodeling. Each type of growth factor has a single major effect on the same intracellular signal transduction pathways through similar types of receptor tyrosine kinases; and it activates both PI3K/AKT and MAPK signaling pathways. Therefore, there is a closely coordinated and organized set of signaling pathways that regulate follicular development in the ovary (12).

Platelet Rich Plasma (PRP), is a biological product that is rich in growth factors. It has been proposed for the purpose of enhancing ovarian function in women who have diminished ovarian reserve, premature ovarian failure, and poor response to assisted reproduction technology (13). However, the available clinical evidence remains heterogeneous. Although its clinical use has become increasingly popular in recent years, the available evidence remains inconsistent and heterogeneous. Consequently, PRP should still be regarded as an experimental intervention in reproductive medicine, and it is not currently incorporated into ESHRE clinical practice guidelines for the management of poor ovarian response (14).

To clarify this question, it is necessary to consider the full range of available evidence. Clinical studies provide information on possible reproductive outcomes, but they rarely identify the molecular pathways responsible for the observed effects. *In vitro* studies and animal models offer more direct mechanistic insights, suggesting that PRP or platelet-derived products may influence follicle survival, granulosa-cell proliferation, angiogenesis, inflammatory modulation, oxidative-stress responses, apoptosis, and mTOR-related signaling. However, these findings are distributed across different experimental systems and have not been fully integrated into a unified mechanistic model of PRP activity in folliculogenesis.

Given the biological complexity of PRP, analysis of individual growth factors in isolation may be insufficient. PRP contains multiple interacting mediators that may act simultaneously on stromal, vascular, immune, and follicular compartments of the ovary. Therefore, a systems-biology approach may provide a more appropriate framework for evaluating whether PRP-associated mediators are mechanistically connected to established regulators of folliculogenesis. In this context, gene mapping, protein–protein interaction network analysis, and pathway enrichment analysis can be used to examine whether PRP-derived factors directly overlap with, or indirectly converge on, molecular pathways known to regulate follicular activation, survival, granulosa–oocyte communication, angiogenesis, and ovarian microenvironmental support.

The present study was designed as a systematic literature-based review combined with an *in silico* computational analysis. First, the available literature was reviewed across three levels of evidence: human clinical studies, *in vitro* studies, and animal models, with the aim of identifying reported biological effects and molecular mediators associated with intraovarian PRP. Second, a folliculogenesis-associated gene map was constructed based on established regulators of human and mammalian follicular development, including oocyte-specific transcription factors, oocyte-derived paracrine mediators, granulosa-cell regulators, gonadotropin and steroidogenic signaling components, angiogenic factors, and intracellular survival pathways. Third, this folliculogenesis gene set was compared with PRP-associated growth factors, receptors, cytokines, and signaling mediators using high-confidence protein–protein interaction and pathway enrichment analysis.

The objective of this combined approach was to determine whether PRP-associated mediators show direct molecular overlap or pathway-level convergence with known regulators of follicular development and ovarian microenvironmental support. Particular emphasis was placed on receptor tyrosine kinase-centered signaling, including FGF/FGFR, EGFR, VEGF, PI3K–AKT, MAPK/ERK, mTOR, and FOXO/PTEN-related pathways. By integrating literature-derived evidence with computational network analysis, this study seeks to provide a mechanistic framework for understanding how PRP may modulate the ovarian microenvironment surrounding existing follicles.

## 2 Materials and methods

### 2.1 Study design

This study was designed as a systematic literature-based review combined with a computational network analysis. The systematic review component aimed to identify clinical, *in vitro*, and animal evidence regarding the effects of platelet-rich plasma (PRP) or platelet-derived products on ovarian function, follicular development, granulosa-cell activity, oocyte competence, and ovarian tissue remodeling. In parallel, the review aimed to extract reported molecular mediators and signaling pathways associated with PRP activity and with established folliculogenesis regulation. The computational component was subsequently used to integrate these literature-derived mediators through gene mapping, protein–protein interaction analysis, and pathway enrichment analysis.

### 2.2 Search strategy

A systematic literature search was performed in PubMed/MEDLINE, Scopus, Web of Science, and Embase from database inception to the date of the final search, without applying any predefined date restriction. The search was restricted to articles published in English. The search strategy combined terms related to platelet-rich plasma and related platelet-derived products, ovarian biology, follicular development, reproductive aging, and molecular signaling. To account for the heterogeneity of terminology used in the literature, the search included the most commonly used terms, such as “platelet-rich plasma” and “PRP,” as well as related nomenclature, including “autologous platelet concentrate,” “platelet lysate,” “platelet concentrate,” “autologous conditioned plasma,” and “platelet-derived products,” where applicable. The following search string was adapted for each database: (“platelet-rich plasma” OR “PRP” OR “autologous platelet concentrate” OR “platelet lysate” OR “platelet concentrate” OR “autologous conditioned plasma” OR “platelet-derived products”) AND (“ovary” OR “ovarian rejuvenation” OR “ovarian reserve” OR “premature ovarian insufficiency” OR “diminished ovarian reserve” OR “poor ovarian response” OR “infertility”) AND (“folliculogenesis” OR “follicular development” OR “granulosa cell” OR “oocyte” OR “primordial follicle” OR “ovarian follicle”) AND (“growth factor” OR “cytokine” OR “PI3K” OR “AKT” OR “MAPK” OR “mTOR” OR “FGF2” OR “VEGF” OR “EGF” OR “PDGF” OR “TGF-beta” OR “bioinformatics” OR “network analysis” OR “pathway enrichment”). Because terminology for platelet-derived preparations is not used consistently across studies, abstracts and, when necessary, full publications were reviewed to determine whether the biological product described was comparable to platelet-rich plasma or represented a relevant platelet-derived preparation. Studies were included when the intervention or product met the review eligibility criteria. Reference lists of eligible articles and relevant reviews were also manually screened to identify additional studies.

### 2.3 Eligibility criteria

#### 2.3.1 Inclusion criteria

Human studies evaluating intraovarian PRP or platelet-derived products in women with diminished ovarian reserve, premature ovarian insufficiency, poor ovarian response or impaired ovarian function; *in vitro* studies assessing PRP, platelet lysate, platelet releasate or platelet-derived products in ovarian follicles, granulosa cells, oocytes or ovarian tissue; animal studies evaluating PRP in models of ovarian injury, ovarian insufficiency, diminished ovarian reserve or follicular dysfunction; and studies reporting molecular mediators or signaling pathways relevant to PRP biology or folliculogenesis.

#### 2.3.2 Exclusion criteria

Non-English articles, conference abstracts without full text, duplicate records, studies unrelated to ovarian biology, studies not involving PRP or platelet-derived products, and articles without clinical, biological or molecular relevance to ovarian function.

### 2.4 Study selection and PRISMA FLOW

The systematic review was conducted and reported in accordance with PRISMA principles. All records retrieved from PubMed/MEDLINE, Scopus, Web of Science, and Embase were imported into a reference-management database, and duplicate records were removed before screening. No predefined publication-date restriction was applied, and only articles published in English were considered eligible.

Titles and abstracts were screened independently by two reviewers to identify studies potentially relevant to intraovarian PRP, platelet-derived products, ovarian function, follicular development, granulosa-cell activity, oocyte competence, ovarian tissue remodeling, and related molecular pathways. Studies considered potentially eligible after title and abstract screening were retrieved in full text and assessed against the predefined inclusion and exclusion criteria. Disagreements regarding study eligibility were resolved by discussion with a senior reviewer.

**Figure 1:**
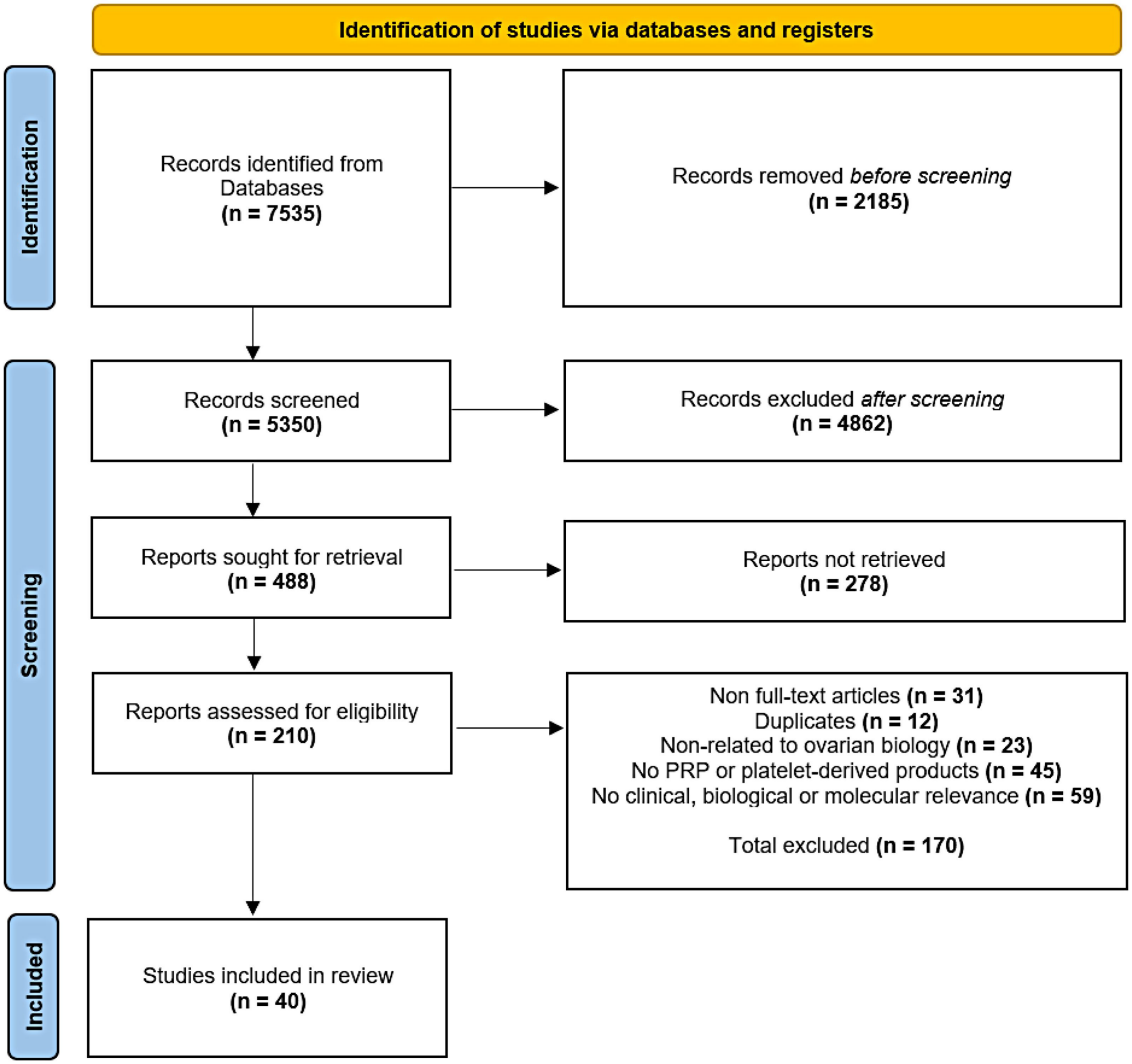
PRISMA studies selection flow diagram. Flow diagram showing the identification, screening, eligibility assessment, and inclusion of studies in this systematic mechanistic review. The bibliographic search was conducted in PubMed/MEDLINE, Scopus, Web of Science, and Embase, from database inception to January 2026, and was restricted to articles published in English. Reference lists of eligible studies and relevant reviews were also manually screened. A supplementary clinical trial registry search was additionally performed in ClinicalTrials.gov, the EU Clinical Trials Register/EudraCT/CTIS, ISRCTN, and the WHO International Clinical Trials Registry Platform; relevant registry records without extractable outcome data are reported separately in Supplementary Table 1.

Reasons for exclusion at the full-text stage were recorded and included absence of PRP or platelet-derived product intervention, lack of relevance to ovarian biology or follicular development, absence of clinical, biological, or molecular outcome data, conference abstract without full text, duplicate publication, non-English publication, or insufficient relevance to the mechanistic objectives of the review. The complete study-selection process is summarized in the PRISMA flow diagram (Figure 1), including the number of records identified, duplicates removed, records screened, full-text articles assessed, full-text articles excluded with reasons, and studies included in the final synthesis.

Eligible studies were classified into three predefined evidence categories: human clinical studies, *in vitro* or cellular studies, and animal or preclinical studies. This classification was used because the included evidence was heterogeneous and because each evidence category contributed differently to the review. Human studies were used mainly to assess clinical outcomes and translational relevance, whereas *in vitro* and animal studies were used primarily to identify mechanistic effects and molecular pathways.

A supplementary clinical-trial registry search was also performed to identify ongoing, unpublished, or registry-only studies evaluating intraovarian or ovarian PRP in women with premature ovarian insufficiency, diminished ovarian reserve, poor ovarian response, low ovarian reserve, or infertility-related ovarian dysfunction. Registry records without posted results or extractable outcome data were not included in the main evidence synthesis but were reported separately in Supplementary Table 1 to improve transparency and document ongoing research activity in the field.

The review combined clinical evidence with mechanistic and computational pathway analysis, the PRISMA framework was applied to the systematic literature component, while the computational gene-set construction and pathway analysis were reported separately in the relevant Methods sections.

### 2.5 Data extraction and evidence classification

Included studies were classified into three evidence categories: human clinical evidence, *in vitro*/cellular evidence, and animal/preclinical evidence. Human studies were used primarily to summarize clinical outcomes and the degree of translational evidence. *In vitro* studies were used to identify direct cellular and tissue-level responses to PRP or platelet-derived products. Animal studies were used to identify preclinical mechanisms of ovarian repair, follicular preservation, angiogenesis, apoptosis regulation, oxidative-stress modulation, and inflammatory responses. This classification was selected because human studies are mainly outcome-oriented, whereas *in vitro* and animal studies provide more direct mechanistic information.

For each included study, the following data were extracted where available: first author, year of publication, study type, experimental model or patient population, indication for PRP use, PRP preparation or platelet-derived product, route and protocol of administration, comparator or control group, main ovarian or reproductive outcomes, molecular markers assessed, signaling pathways reported, and main limitations. For human studies, extracted outcomes included AMH, AFC, oocyte yield, embryo development, pregnancy, and live birth where reported. For *in vitro* and animal studies, extracted outcomes included follicle survival, granulosa-cell proliferation, oocyte competence, follicular atresia, angiogenesis, apoptosis, oxidative stress, inflammatory modulation, mitochondrial function, and pathway activation.

### 2.6 Risk-of-bias assessment

Risk of bias was assessed according to study design. Because the included evidence consisted of randomized clinical trials, observational human studies, case reports, *in vitro* studies, and animal experiments, a single uniform assessment tool was not considered appropriate. Instead, study quality was evaluated using design-specific criteria.

For randomized clinical trials, methodological quality was assessed using domains derived from the Cochrane Risk of Bias 2 (RoB 2) framework, including adequacy of randomization, deviations from intended intervention, missing outcome data, outcome measurement, and selective reporting. The three randomized controlled trials included in this review—Barrenetxea et al. (15), Herlihy et al. (PROVA trial) (16), and Yahyaei et al. (17)—were considered the highest level of available clinical evidence. However, because complete methodological details such as allocation concealment, protocol registration, and blinding procedures were not consistently reported in all studies, these trials were interpreted with some caution when evaluating clinical efficacy.

Animal studies were assessed descriptively according to the ARRIVE 2.0 Essential 10 criteria, including study design, sample size, inclusion and exclusion criteria, randomization, blinding, outcome measures, statistical methods, experimental animals, experimental procedures, and reporting of results. Across the included preclinical studies [Dehghani et al. (18), Ozcan et al. (19), Budak et al. (20), Vural et al. (21), Ahmadian et al. (22), Anvari et al. (23), Zhao et al. (24), Cozzolino et al. (25), Fotoohi-Ardakani et al. (26), and El Bakly et al. (27)], reporting was generally adequate for study design, experimental procedures, outcome measures, animal model description, and results reporting. However, important methodological safeguards such as sample-size justification, explicit randomization procedures, blinding of outcome assessment, and predefined exclusion criteria were inconsistently reported.

*In vitro* studies, including Pazoki et al. in 2015 and 2016, were assessed qualitatively based on the presence of appropriate controls, reproducibility of experimental conditions, characterization of platelet-derived products, and objective measurement of biological endpoints.

Risk-of-bias assessment was not used to exclude studies from the review but rather to guide interpretation of the evidence. Greater weight was assigned to randomized human studies when evaluating clinical efficacy, whereas mechanistic conclusions were primarily supported by well-controlled preclinical and molecular studies. Overall, the available evidence suggests biological plausibility for PRP-mediated ovarian effects, although heterogeneity in PRP preparation protocols and limitations in study design reduce the strength of definitive clinical conclusions.

### 2.7 Molecular mediator extraction and gene-set construction

Molecular mediator extraction was performed to generate two predefined literature-derived gene sets for downstream computational analysis: a PRP-associated gene set and a folliculogenesis-associated gene set. The purpose of this step was not to include all molecules reported in ovarian biology or PRP research, but to construct two biologically interpretable gene frameworks that could be compared at the level of direct gene overlap, protein–protein interaction connectivity, and pathway-level convergence.

Molecular mediator extraction was performed to generate two predefined literature-derived gene sets for downstream computational analysis: a PRP-associated gene set and a folliculogenesis-associated gene set. These gene sets were generated through targeted molecular mapping informed by the systematic review, but they were not restricted exclusively to the papers included in the systematic review. The systematic review was first used to identify the main biological themes relevant to PRP and ovarian biology, including angiogenesis, inflammation regulation, extracellular-matrix remodeling, stromal repair, granulosa-cell survival, apoptosis regulation, oxidative-stress modulation, growth-factor signaling, and follicular development. Additional mechanistic papers and reviews were then consulted when they provided specific molecular information required for gene-set construction.

For the PRP-associated gene set, molecules were selected if they were reported as components of PRP or platelet-derived products, or as mediators of PRP-related biological activity. These included platelet-derived growth factors, cytokines, chemokines, angiogenic mediators, receptors, extracellular vesicle-associated mediators, extracellular-matrix remodeling molecules, and intracellular signaling components involved in tissue repair, inflammation regulation, angiogenesis, stromal remodeling, cell survival, or proliferation. Studies were not used for PRP gene extraction if they reported only clinical outcomes without molecular information, described PRP effects without identifying specific mediators, or referred only to broad pathway terms that could not be assigned to defined genes or proteins. The final PRP-associated gene set used for computational analysis is presented in Table 1.

**Table 1:** Platelet-rich plasma-associated growth factors, receptors, and molecular mediators included in the computational gene set. The table lists the principal PRP-related proteins identified from the literature and the corresponding standardized human gene symbols used for subsequent gene-overlap, protein–protein interaction, and pathway-enrichment analyses. PRP, platelet-rich plasma.

PRP growth factor/mediator	Gene symbol
Fibroblast growth factor 2	FGF2
Epidermal growth factor	EGF
Transforming growth factor beta 1	TFGB1
Platelet-derived growth factor subunit A	PDGFA
Platelet-derived growth factor subunit B	PDGFB
Vascular endothelial growth factor A	VEFGA
Insulin-like growth factor 1	IGF1
Insulin-like growth factor 2	IGF2
FGF receptor 1-2-3-4	FGFR1-FGFR2-FGFR3-FGFR4
PDGF receptor alpha-beta	PDGFRA-PDGFRB
VEGF receptor 1-2	FLT1-KDR
IGF1 receptor 1-2	TGFBR1-TGFBR2

For the folliculogenesis-associated gene set, molecules were selected if they had an established role in human or mammalian follicular development according to eligible folliculogenesis literature and targeted mechanistic sources. These included genes involved in oocyte-specific transcriptional programming, oocyte-derived paracrine signaling, primordial follicle activation and dormancy, granulosa-cell differentiation, gonadotropin and steroidogenic signaling, angiogenesis, intracellular survival signaling, and follicular growth regulation. Sources were not used for folliculogenesis gene extraction if they described follicular development only in broad biological terms without identifying specific molecular regulators. The final folliculogenesis-associated gene set is presented in Table 2.

**Table 2:** Gene mapping in folliculogenesis.

Gene name	Role in folliculogenesis	References
Core oocyte regulators		
GDF9	GDF-9 is an oocyte-derived growth factor that promotes granulosa cell proliferation and early follicle development, thereby supporting follicular growth and oocyte developmental competence	Sanfins et al. (28), Otsuka et al. (29), Fountas et al. (30)
BMP-15	BMP-15 is an oocyte-secreted regulator that modulates granulosa cell differentiation and FSH responsiveness, playing a critical role in follicle maturation and cumulus–oocyte complex function.	Sanfins et al. (28, 30–33)
FIGLA	FIGLA is an oocyte-specific transcription factor essential for primordial follicle formation and early oocyte differentiation. Loss-of-function models demonstrate severe follicular depletion and infertility, highlighting its role as a master regulator of early folliculogenesis.	Huntriss et al. (34–36)
NOBOX	NOBOX is an oocyte-specific homeobox transcription factor that regulates oocyte gene expression programs essential for early folliculogenesis, primordial follicle activation, and maintenance of the ovarian reserve.	Huntriss et al. (34, 37, 38)
SOHLH 1	Transcription factor required for oocyte differentiation and primordial follicle formation.	Wang et al. (29), Shin et al. (39)
SOHLH 2	Early germline transcription factor that initiates the oocyte differentiation transcriptional program.	Zhang et al. (9, 40)
Primordial follicle activation/survival (PI3K pathway)		
PTEN	PTEN (Phosphatase and tensin homolog) is a critical suppressor of the PI3K/Akt pathway, acting as a crucial molecular brake that maintains ovarian follicle dormancy and prevents premature exhaustion of the primordial follicle pool.	Goto et al. (41, 42)
FOXO3	FOXO3 is a key transcription factor that maintains primordial follicle dormancy and regulates the activation of the ovarian follicle reserve.	Pelosi et al. (43–46)
PIK3CA	PIK3CA (Phosphatidylinositol-4,5-bisphosphate 3-kinase catalytic subunit alpha) plays a critical, central role in folliculogenesis, specifically in regulating the activation and growth of primordial follicles.	Kim et al. (47, 48)
AKT1	It facilitates the transition of primordial follicles from dormancy to growth and controls oocyte maturation.	Longobardi et al. (49–51)
MTOR	mTORC1 acts as a key metabolic sensor in oocytes. Activation of mTOR, often via TSC1/TSC2/PTEN regulation, promotes the initiation of growth in dormant follicles.	Chen et al. (52, 53)
Granulosa cell differentiation and follicle structure		
FOXL2	Acts to maintain the pool of resting primordial follicles; its absence leads to massive, premature activation of primordial follicles followed by rapid follicular depletion and atresia. Essential for the structural transition of squamous pre-granulosa cells to cuboidal granulosa cells in primary follicles.	Kuo et al. (54–57)
GATA4	GATA4 is a critical transcription factor in follicular development, with its absence leading to severely impaired folliculogenesis, increased atretic follicles, and infertility.	Efimenko et al. (58, 59)
GATA6	GATA6 is involved in regulating genes related to steroidogenesis, cell division, and extracellular matrix organization within the ovarian follicle. Acting alongside GATA4 in ovarian granulosa cells, is essential for folliculogenesis, regulating follicular assembly, growth, and luteinization	Bennett et al. (60)
NR5A1	It acts as a key regulator of steroidogenic enzyme-encoding genes (e.g., CYP11A1, CYP19A1, HSD17B1, HSD3B2), with expression levels typically increasing as follicles progress in growth.	Song et al. (61, 62)
Gonadotropin and hormone signaling		
FSHR	It is crucial for rescuing follicles from apoptosis (programmed cell death) and promoting the growth of Graafian follicles.	Achrekar et al. (63, 64)
LHCGR	LHCGR mediates LH signaling in granulosa and theca cells, enabling late follicular maturation, ovulation, and steroidogenic activation essential for successful folliculogenesis.	Gulappa et al. (65–67)
ESR1	Esr1 is crucial for the progression of follicles to the pre-ovulatory stage. Deficiency in Esr1 (as seen in knockout mice) leads to an arrest in folliculogenesis, with follicles often developing into large, hemorrhagic cysts rather than ovulating.	He et al. (68–71)
ESR2	ESR2 maintains the ovarian reserve by limiting the rate of activation of primordial follicles. Disruption of ESR2 leads to excessive activation, causing premature ovarian exhaustion.	Chauvin et al. (71–73)
AR	AR activation by androgens (testosterone, DHT) is essential for the transition of primordial follicles to primary follicles.	Kimura et al. (74–76)
Steroidogenesis		
CYP11A1	CYP11A1 ensures that GCs have the capacity to produce estrogen and, later, progesterone. It is essential for the transformation of GCs into granulosa lutein cells, which produce large amounts of progesterone.	Fouikar et al. (77, 78)
HSD3B1	HSD3B1 is a key steroidogenic enzyme that catalyzes the conversion of pregnenolone to progesterone and other *Δ*4 steroids, thereby supporting granulosa cell steroidogenesis and the hormonal environment required for follicular growth and maturation during folliculogenesis.	Orozco-Galindo et al. (3, 79, 80)
HSD17B1	HSD17B1 is a key steroidogenic enzyme that catalyzes the conversion of estrone to estradiol in granulosa cells, thereby supporting estrogen production required for follicular growth, granulosa cell function, and oocyte maturation during folliculogenesis.	Penning (81, 82)
CYP19A1	CYP19A1 (aromatase) catalyzes the conversion of androgens to estrogens in granulosa cells, thereby generating the estradiol-rich follicular environment required for granulosa cell proliferation, follicle maturation, and acquisition of oocyte developmental competence during folliculogenesis.	Hashemain et al. (83, 84)
STAR	STAR (Steroidogenic Acute Regulatory Protein) mediates the transport of cholesterol into mitochondria, representing the rate-limiting step of steroid hormone synthesis and thereby enabling steroidogenesis required for normal follicular development and maturation during folliculogenesis.	Galano et al. (85, 86)
Follicle growth factors		
BMP4	BMP4 facilitates the activation of primordial follicles and their transition to primary follicles, with high expression throughout all stages of follicle development.	Fountas et al. (30, 87, 88)
FGF2	FGF2 acts as a potent recruiter of primordial follicles into the growing pool. Studies in bovine and rat models show that FGF2 treatment significantly increases the percentage of developing follicles	Kanke et al. (89–91)
IGF1	IGF1 promotes folliculogenesis by enhancing granulosa cell proliferation, steroidogenesis, and survival, while synergistically amplifying gonadotropin signaling required for follicle growth, selection, and maturation.	Adashi et al. (92, 93)
TGFR1	TGFBR1-mediated signaling, specifically by ligands like TGF-β1, supports follicle development, promotes granulosa cell proliferation, and influences follicular maturation, particularly as follicles transition to the antral stage.	Wang et al. (94–96)
VEGFA	VEGFA acts as a survival factor for granulosa and theca cells, preventing apoptosis (programmed cell death), which is crucial for preventing follicle atresia (demise)	McFee et al. (97–99)
Developmental signaling pathways- WNT pathway		
WNT3A, WNT5A,WNT4, WNT6, FZD1, LRP11	Canonical WNT signaling is essential for the transition of squamous pre-granulosa cells to cuboidal granulosa cells (GCs), enabling the initiation of follicle growth. WNT ligands, often acting in an autocrine manner, drive the differentiation of pre-granulosa cells into mature GCs. The WNT/\(\beta \)-catenin pathway regulates follicular progression from the preantral to the antral stage, with \(WNT2\) and \(WNT4\) expressed specifically in GCs of these follicles.	Hsieh et al. (10, 100–103)
Developmental signaling pathways- hedgehog pathway		
GLI1, GLI2, GLI3, SMO, PTCH1	Hh signaling, often in collaboration with other signaling factors like insulin-like growth factor (IGF1), supports granulosa cell proliferation and overall follicle growth.	Luo et al. (102, 104, 105)
Granulosa–oocyte communication/ovulation signaling		
EGFR	EGFR signaling is vital for the transition from the primary to the secondary follicle stage (follicle activation). Disruption of egfra (a form of EGFR) in studies has been shown to cause follicular arrest, leading to infertility.	Wu et al. (106)
AREG	AREG is essential for cumulus expansion, which is necessary for oocyte maturation. Studies show that adding AREG to *in vitro* maturation (IVM) media significantly increases the rate of MII oocyte maturation.	Fang et al. (107)
EREG	EREG functions as a key EGF-like growth factor that transmits the ovulatory signal from LH to the rest of the follicle	Zhao et al. (108)
BTC	BTC is rapidly induced in preovulatory follicles by LH/hCG. It is crucial for promoting cumulus expansion, oocyte maturation, and the transformation of granulosa cells into luteal cells (luteinization).	Li et al. (109)
Anti-Müllerian axis		
AMH—AMHR2	AMH limits the initiation of growth (initial recruitment) of primordial follicles into the primary follicle stage. This prevents premature exhaustion of the ovarian reserve. In later stages, AMH reduces the sensitivity of growing preantral and small antral follicles to follicle-stimulating hormone (FSH). This action prevents too many follicles from being selected for growth at the same time, thereby reducing FSH-dependent development.	Iwase et al. (110, 111)
Oocyte meiosis and maturation competence		
PDE3A	PDE3A plays a key role in folliculogenesis by regulating oocyte meiotic arrest and resumption through control of intracellular cAMP levels, thereby coordinating oocyte maturation and follicular development.	Gershon et al. (112)
CDC25B, CDC25C	CDC25B plays a critical role in folliculogenesis by activating CDK1 and the maturation-promoting factor (MPF), thereby enabling oocyte meiotic resumption and progression of oocyte maturation within the follicle.	Xie et al. (113–116)
AURKA, AURKB, AURKC	Aurora kinases A, B, and C (AURKA, AURKB, AURKC) are critical regulators of meiotic cell division during oocyte maturation within the process of folliculogenesis, primarily ensuring proper chromosome segregation and spindle assembly. While AURKA is crucial for spindle assembly, AURKB and AURKC (often acting as the Chromosomal Passenger Complex—CPC) are essential for regulating chromosome alignment and kinetochore-microtubule attachments during meiosis I.	Rengaraj and Han (117)
Cell survival/atresia regulation		
BCL2, BCL2L1, BCL2L2, BCL2L10, BCL2L11, BCL2L14	Bcl-2 overexpression increases the number of primordial follicles at birth. However, this excess is often not maintained into adulthood, suggesting an ovarian “census” mechanism that removes extra follicles.	Albamonte et al. (118, 119)
Cytoskeleton/cell signaling modulators		
ARHGAP9, ARHGAP20, ARHGAP21, ARHGAP31, ARHGAP36, ARHGAP44	ARHGAP genes contribute to folliculogenesis by regulating Rho GTPase signaling pathways that control cytoskeletal dynamics, granulosa cell proliferation, and follicular structural remodeling essential for follicle growth and oocyte maturation.	Li et al. (120, 121)

All extracted molecules were standardized to official human gene symbols using NCBI Gene. When animal or *in vitro* studies reported non-human gene names, the corresponding human orthologs were identified and used for computational analysis where appropriate. Molecules described only as broad pathway terms, biological processes, or nonspecific protein families were excluded from the computational gene sets if they could not be assigned to a specific gene or protein. However, such terms were retained in the narrative synthesis when they were biologically relevant to PRP activity or folliculogenesis.

All extracted molecules were standardized to official human gene symbols using NCBI Gene. When animal or *in vitro* studies reported non-human gene names, the corresponding human orthologs were identified and used for computational analysis where appropriate (Supplementary Table 2). Molecules described only as broad pathway terms, biological processes, or nonspecific protein families were excluded from the computational gene sets if they could not be assigned to a specific gene or protein. However, such terms were retained in the narrative synthesis when they were biologically relevant to PRP activity or folliculogenesis.

### 2.8 Protein–protein interaction network analysis

Protein-protein interaction (PPi) networks of Homo sapiens were created from data in the STRING database (122). First, PPi networks were separately created for the gene set associated with folliculogenesis, and the gene set associated with PRP. Then a single PPi network was created to combine both gene sets; this network allows evaluation of direct and indirect molecular overlap between mediators of PRP, and regulators of folliculogenesis.

A high confidence threshold of ≥0.7 was used for an “interaction” to ensure that all connections had at least a high degree of support. Sources of information for active interactions included experimental validation, curated pathways and expression patterns. All non-experimental and indirect sources of information, including text mining, co-localization, gene fusion and co-occurrence, were excluded to avoid association by frequency alone within literature references. Also no other genes than those identified through systematic review were included as first shell or second shell interactors, thereby limiting the complexity of the network to the pre-defined list of genes. Nodes separated into sub-networks were kept to allow for tabular analysis, however they can also be eliminated in graphical representations for improved visualization. The output files of each PPi network were saved in two file types, TSV and SVG, to facilitate further analysis and graph construction.

The whole network was examined in order to determine which central actors (i.e., “mediators”) are responsible for converging on common pathways. Parameters used to describe this network structure included degree centrality, betweenness centrality, closeness centrality, and cluster coefficient. Genes that ranked high across one or more of these centrality parameters were termed “hub” genes.

Functional enrichment of all genes sets, as well as the total network, has been done. The enrichment was determined through GO-BP (Gene Ontology Biological Processes), KEGG (Kyoto Encyclopedia of Genes and Genomes) pathway and Reactome pathway. Statistical significance of enrichment was analyzed by a hypergeometric test vs. the whole genome; multiple testing corrections were performed through an application of False Discovery Rate. A statistical enrichment significance of *p*-Value adjusted by the FDR < .05 is required in order for a pathway to be called statistically enriched. An analysis of relevance to known follicular developmental processes such as cell survival, proliferation, angiogenesis, growth factor signaling, granulosa-oocyte communication, steroidogenesis and primordial follicle activation was conducted.

These pathways were prioritized because they represent the principal mechanistic interfaces between PRP-associated growth-factor signaling and established regulators of follicular development. In particular, receptor tyrosine kinase, PI3K–AKT, MAPK/ERK, mTOR, FOXO/PTEN, VEGF, EGFR, FGFR, and TGF-β/BMP-related pathways are directly relevant to granulosa-cell survival and proliferation, angiogenesis, apoptosis regulation, oxidative-stress responses, primordial follicle activation or dormancy, steroidogenic activity, and oocyte–granulosa-cell communication. Their selection was therefore guided by both the biological themes emerging from the systematic review and their known relevance to ovarian follicular biology.

To assess how mechanisms are being used across two different groups (PRP-associated mediators, and those related to folliculogenesis), we identified enriched pathways from each group. We then determined if there were enriched pathways common to both groups; these pathways could be pathways that had a significant representation in both datasets, and/or pathways that included genes associated with PRP, and/or genes involved in folliculogenesis at function-related upstream and/or downstream positions.

The degree of pathway overlap between the two groups was assessed through an evaluation of the number of overlapping pathways, the proportion of overlap (i.e., the ratio of the size of the intersection over the size of the union) and where applicable, Jaccard similarity coefficient. A hypergeometric test, or when feasible a permutation-based comparison, was utilized to evaluate whether the amount of pathway overlap seen was greater than would be expected by random chance.

We employed this method to differentiate overlapping in terms of individual gene presence vs. pathway level mechanism usage. Mechanism overlapping via gene directly corresponds to overlapping genes in the dataset. Mechanisms that converge correspond to separate genes contributing to the same signaling pathway or a biologically-coherent set of related pathways.

The genes associated with the PRP-mediated set and the folliculogenesis set were mapped onto KEGG pathway maps through use of the KEGG mapper. A number of biological relevant and statistically enriched from previous analyses KEGG pathways were chosen for inclusion based on their relevance to biological processes involved in follicular growth/development.

The genes that were mapped onto each of the selected KEGG pathways are represented by colors indicating either PRP-mediated genes, folliculogenesis mediated genes or both; this visualization will allow us to see if we can position genes related to PRP as “upstream” of other cellular survival/proliferation mechanisms and/or place genes related to folliculogenesis downstream of or within cell-type specific regulatory modules.

To synthesize the findings from the Systematic Review and Computational Analysis which provided narrative and mechanistic integration, we analyzed PRP-associated molecular mediators and folliculogenesis regulators in order to find if they interacted as coherent networks or shared biological pathways using computational methods. We interpreted our data at the pathway level of convergence instead of simply looking at overlapping genes. This decision was made due to the complexity of PRP with respect to multiple growth factors and cytokines that can modulate signaling environment, thus providing a more accurate representation of how PRP will impact the oocytes compared to replacing their specific transcriptional programs.

### 2.9 Statistical analysis

Descriptive synthesis was used to summarize the clinical, *in vitro*, and animal studies included in the systematic review because of the substantial heterogeneity in study design, patient or experimental population, PRP preparation, comparator groups, and reported outcomes. No meta-analysis was performed. For the computational component, functional enrichment analyses were performed for the PRP-associated gene set, the folliculogenesis-associated gene set, and the combined network. Gene Ontology Biological Process, KEGG, and Reactome pathway enrichment were assessed using a hypergeometric test against the whole-genome background. Multiple-testing correction was performed using the false discovery rate method, and pathways with FDR-adjusted *p*-values < 0.05 were considered statistically enriched.

Pathway overlap between the PRP-associated and folliculogenesis-associated gene sets was evaluated by comparing the number and proportion of shared enriched pathways. Where applicable, overlap was summarized using the Jaccard similarity coefficient, and enrichment of pathway overlap was assessed using a hypergeometric or permutation-based approach. Protein–protein interaction networks were generated using STRING with a high-confidence interaction score threshold of ≥0.700. Network topology was assessed descriptively using degree centrality, betweenness centrality, closeness centrality, and clustering coefficient to identify hub genes and pathway convergence. The computational analyses were interpreted as hypothesis-generating and were not considered direct evidence of pathway activation in ovarian tissue.

## 3 Results

### 3.1 Systematic review

#### 3.1.1 Human evidence

The majority of existing clinical research evaluating the effects of intra-ovarian platelet-rich plasma (PRP) on ovarian function has been conducted as observational studies (123–135) without control groups, cohort studies (15, 129–135) and few randomized controlled trials (16, 17, 136). Studies on human subjects have primarily looked at women diagnosed with poor ovarian reserve (137, 138); diminished ovarian reserve (15, 17, 127, 130, 132, 136, 139–141); anovulatory dysfunction (134) or history of poor outcomes in IVF (135, 142) (Table 3).

**Table 3:** Human studies.

Study	Year	Design	Population/Indication	Findings	AFC	Outcome
Sills et al. (143)	2018	Case series	Poor-prognosis IVF patients receiving intraovarian calcium gluconate–activated autologous PRP	mature oocyte retrieval/blastocyst formation	Not reported	Mature oocytes were retrieved and blastocyst formation was achieved after PRP
Pantos et al. (39)	2019	Case series	Menopausal and prematurely menopausal women/POF after failed IVF attempts	PRP was followed by apparent restoration of ovarian activity and natural conception.	Not reported	Natural conception occurred within 2–6 months, resulting in ongoing complication-free clinical pregnancies.
Stojkovska et al. (137)	2019	Clinical study	Poor ovarian responders undergoing IVF/ICSI with low-dose stimulation	PRP group showed a trend toward higher implantation, clinical pregnancy, and live birth rates, but differences were not statistically significant.	Not reported	Clinical pregnancy 33.33% vs. 10.71%; live birth 40.00% vs. 14.29% in PRP vs. control groups.
Melo et al. (138)	2020	Clinical study	Women with low ovarian reserve undergoing fertility treatment	PRP improved ovarian reserve markers before ART compared with no intervention.	AFC significantly improved at 3 months after PRP.	Biochemical and clinical pregnancy rates were higher in the PRP group; further evidence needed for pregnancy outcomes.
Hsu et al. (128)	2020	Case report	Woman with premature ovarian insuffuciency	Pregnancy/live birth	Not reported	Reported live birth after intraovarian PRP, very low-level evidence because it is a single case.
Petryk and Petryk (131)	2020	Clinical study	Women with low ovarian reserve	AMH, FSH/LH, follicular response, pregnancy outcomes	Improvement in ovarian reserve not statistically significant	Reported improvement in ovarian reserve markers and reproductive outcomes in some women with low ovarian reserve after intraovarian PRP; uncontrolled observational evidence.
Sills et al. (144)	2020	Clinical study	Women with low ovarian reserve	IVF-related outcomes	Not reported	Reported spontaneous conception and term delivery after intraovarian autologous platelet growth-factor concentrate in a woman over 40; case-level evidence.
Sabouni et al. (145)	2021	Case report	Women with low ovarian reserve	AMH, pregnancy outcome	Not reported	Reported AMH increase and follicular development after PRP in a patient with ovarian insufficiency; case-report evidence.
Navali et al. (146)	2022	Clinical study	Low ovarian reserve	Intraovarian autologous PRP significantly increased oocyte number, embryo number, and estradiol levels.	Not reported	Suggested potential benefit for achieving conception with autologous oocytes; live birth impact not established.
Tülek et al. (127)	2022	Clinical study	Poor responders	AFC, pregnancy outcome	Not reported	Reported improved IVF-related parameters in some poor responders and women with premature ovarian insufficiency after intraovarian PRP; observational evidence.
Cakiroglu et al. (132)	2022	Clinical study	Low ovarian reserve	AFC, pregnancy outcome	Evaluated ovarian reserve parameters including AFC and IVF outcomes	Reported improvement in ovarian reserve parameters after PRP; observational design limits causal inference.
Merhi et al. (147)	2022	Clinical study	Infertility	AMH, AFC, pregnancy outcome	Improvement in ovarian reserve not statistically significant	Reported possible improvement in blastocyst euploidy rate after intraovarian PRP in women undergoing IVF; preliminary clinical evidence.
Hosseinisadat et al. (133)	2023	Clinical study	Low ovarian reserve	Embryo quality, pregnancy outcome	Evaluated ovarian reserve markers including AFC	Reported improvement in ovarian reserve markers after intraovarian PRP in women with poor ovarian reserve; before-and-after design.
Najafian et al. (148)	2023	Clinical study	Poor responders	AMH, AFC, pregnancy outcome	Improvement in ovarian reserve not statistically significant	Reported favorable fertility outcomes after direct intraovarian PRP injection in poor ovarian responders; quasi-experimental evidence.
Tanha et al. (149)	2023	Clinical study	Low ovarian reserve	Safety, ovarian response	Not reported	Reported possible improvement in ovarian response/reserve markers after intraovarian PRP in poor ovarian response; preliminary clinical evidence.
Sills et al. (142)	2023	Clinical study	Infertility	Oxidative stress, apoptosis, hypoxia, metabolism, ovarian signaling	Useful mechanistic study; supports biological plausibility rather than clinical efficacy.	Discussed condensed platelet cytokines for infertility/menopause and proposed biological mechanisms; not primary efficacy evidence.
Sills et al. (27)	2023	Clinical study	Infertility	mTOR, ovarian reserve signaling, follicular activation	Supports mechanistic rationale linking platelet cytokines with mTOR/follicular signaling; not primary efficacy proof.	Proposed combined intraovarian platelet cytokines plus low-dose pulsed rapamycin as a mechanistic strategy targeting diminished ovarian reserve; hypothesis/protocol-level evidence.
Barrenetxea et al. (17)	2024	Clinical study	Poor responders	AFC, pregnancy outcome	Evaluated ovarian reserve markers including AFC	Randomized trial reported increased number of retrieved oocytes after PRP but no improvement in blastocyst quality.
Herlihy et al./PROVA trial (136)	2024	Clinical study	Poor responders	AFC, pregnancy outcome	Included AMH and AFC among ovarian reserve outcomes	Randomized trial found no improvement in mature oocyte yield, blastocyst outcomes, euploid blastocysts, AMH, or AFC after PRP.
Devenutto et al. (130)	2024	Clinical study	Poor responders	AMH, pregnancy outcome	Not reported	Reported favorable ART outcomes after intraovarian PRP in poor responders compared with previous cycle; non-randomized evidence.
Potiris et al. (134)	2024	Clinical study	Women with anovulatory cycles	AFC, pregnancy outcome	Not reported	Reported preliminary improvement in ovarian function markers after intraovarian PRP in anovulatory infertility; prospective cohort evidence.
Yu et al. (135)	2024	Clinical study	IVF patients with previous poor embryo quality	Pregnancy outcome	Not reported	Reported improved blastocyst yield and quality after intraovarian PRP in IVF patients; clinical evidence requiring cautious interpretation.
Molinaro et al. (15)	2025	Clinical study	Low ovarian reserve	AFC. AMH, pregnancy outcome	In the POI cohort, AFC increased from baseline around 1 to approximately 2 follicles after PRP, but IVF/reproductive outcomes did not improve	Important because it shows AFC can increase without meaningful IVF benefit.
Li et al. (120)	2025	Clinical study	Poor responders	AFC, pregnancy outcome	Not reported	Compared single vs. double intraovarian PRP injections and reported protocol-dependent ovarian-response differences in poor ovarian responders.
Nulianti et al. (126)	2025	Clinical study	Premature ovarian insufficiency	AMH, pregnancy outcome	Not reported	Case series reported AMH improvement and ovarian response in POI patients undergoing IVF, with limited case-level evidence.
Yahyaei et al. (16)	2026	Clinical study	Low ovarian reserve	AFC	AFC was significantly higher in the PRP group, *p* = 0.001	Recent evidence suggesting AFC improvement, but it should be interpreted with the full design and risk-of-bias context.
Sfakianoudis et al. (123)	2019	Clinical study	Poor responders	AMH, pregnancy outcome	Not reported	Reported reduced FSH, increased AMH, improved embryo quality, and natural conception in a small poor-responder case series.
Sfakianoudis et al. (125)	2020	Clinical study	Poor responders	AMH, pregnancy outcome	Not reported	Reported preliminary ovarian-function reactivation in selected women with POI, perimenopause, menopause, or poor ovarian response.

Clinical evidence early on were based mostly on individual cases. For example, Hsu et al. and Pantos et al. reported a live birth after administration of intra-ovarian PRP plus gonadotropins to a woman who had premature ovarian failure (39, 128). Sills et al. set as an extra parameter the age and reported spontaneous conception and term delivery after intra-ovarian administration of autologous platelet-derived growth factor concentrate in a woman older than 40 (124). Additionally, Sills et al. evaluated intraovarian injection of activated autologous platelet-rich plasma in poor-prognosis IVF patients and reported post-treatment changes in serum anti-Müllerian hormone levels, further supporting its inclusion among early uncontrolled clinical reports (144). Sabouni et al. also documented increases in AMH and follicular activity after PRP administered to a woman with ovarian insufficiency (145).

Subsequently, numerous observational clinical studies evaluated various markers of ovarian reserve and/or IVF outcomes after intra-ovarian PRP administration. Petryk and Petryk observed improvements in ovarian reserve markers and reproductive outcomes in women with low ovarian reserve although the improvement in ovarian reserve was not statistically significant (131). Tülek and Kahraman observed improvements in IVF related outcomes after intra-ovarian PRP administration in women with premature ovarian failure or poor responders (127). Similar studies reported better numbers of mature oocytes and embryos after intraovarian PRP administration (137, 143, 146). Cakiroglu et al. studied a large group of women with low ovarian reserve or poor responses during IVF cycles and noted improvements in ovarian reserve and IVF outcomes after intra-ovarian PRP administration. Specifically, they measured antral follicle count (AFC), which can serve as a marker of ovarian reserve, and evaluated reproductive outcomes (132). Melo et al. (138) reported improved AFC and ART outcome without emphasizing on pregnancy and birth rates. Hosseinisadat et al. also documented improvements in markers of ovarian reserve after intra-ovarian PRP administration in women with poor responses (133). Najafian et al. documented positive fertility outcomes after intra-ovarian PRP administration directly injected into the ovaries of poor responders however the improvement in ovarian reserve was not statistically significant (148).

Other clinical studies examined IVF laboratory outcomes. Merhi et al. observed what appeared to be an improvement in the proportion of euploid blastocysts after intra-ovarian PRP administration in women undergoing IVF although there was no statistical significance regarding improvement in ovarian reserve (147). Yu et al. observed improved blastocyst yields and quality after intra-ovarian PRP administration to IVF patients previously producing embryos with lower quality (135). These studies indicate that intra-ovarian PRP may affect oocyte/embryo competence but the data remain preliminary and require careful consideration given study variability and limited randomization.

There are few randomized controlled studies available examining the effectiveness of intra-ovarian PRP for improving IVF outcomes. The most well-known randomized controlled study was performed by Barrenetxea et al. They demonstrated that intra-ovarian PRP increased the number of oocytes obtained from women considered poor responders to IVF treatments but did not affect blastocyst quality (17). In contrast, the PROVA randomized controlled trial, conducted by Herlihy et al., failed to demonstrate a difference between the group receiving intra-ovarian PRP vs. placebo in terms of mature oocyte yields, blastocyst yields/euploidy status, AMH levels or AFC (136). Overall, these results would appear to indicate that intra-ovarian PRP does not reliably improve important IVF outcome measures especially when evaluated within a randomized controlled setting.

AMH was assessed in multiple studies however results varied greatly. Several studies evaluated AFC as part of assessing ovarian reserve or ovarian responsiveness. Cakiroglu et al., Hosseinisadat et al., Barrenetxea et al., Herlihy et al., Molinaro et al., Li et al., Potiris et al., and Yahyaei et al. all identified AFC as a variable they used to assess aspects of ovarian reserve/response (15–17, 129, 132–134, 136). Molinaro et al. indicated that AFC increased by 1 follicle post-intra-ovarian PRP treatment in a POI subgroup from 1 follicle pre-treatment to 2 follicles post-treatment however the change did not result in measurable differences in IVF/reproductive benefits (15). Additionally, Yahyaei et al. reported significantly higher AFC in the PRP-treated group compared with the non-treated control group (17). On the other hand, the PROVA trial showed no significant difference between groups regarding AFC indicating that AFC changes after PRP are not consistently seen across studies (136).

Finally, fewer studies explored whether there were biologic mechanisms explaining how PRP might exert beneficial effects on ovarian activity. Sills et al. published a review outlining various biologic mechanisms through which oxidative stress, apoptosis, hypoxia, metabolism and ovarian signaling might influence or contribute to decreased ovarian activity resulting from aging/menopause (142). Another article by Sills et al. proposed combining intra-ovarian platelet cytokine stimulation with low dose pulsed Rapamycin as a potential treatment approach to stimulate mTOR mediated pathways involved in maintaining/activating ovarian reserves/follicles (27). These articles supported biologic plausibility of PRP-induced signals however they provided no primary evidence supporting clinical efficacy.

In summary, while many human clinical studies demonstrated that intra-ovarian PRP may lead to improved activity in selected women's ovaries (e.g., AMH, AFC, follicular response, oocyte yield, embryonic development and occasionally pregnancy/live birth) the clinical evidence appears quite heterogeneous/methodologically weak. Thus, while many observational/case-based studies report positive outcomes for women treated with intra-ovarian PRP randomized-controlled evidence supports fewer such conclusions.

#### 3.1.2 *In vitro* evidence

*In vitro* and cellular studies provided mechanistic support for a direct effect of PRP or platelet-derived products on ovarian follicles, granulosa cells, and ovarian tissue. These studies used heterogeneous models, including isolated human primordial and primary follicles, mouse and murine preantral follicle culture, bovine ovarian sections, granulosa-cell culture, ovarian tissue molecular models, and xenotransplanted human ovarian tissue (Table 4).

**Table 4:** *In vitro* studies.

Study (*in vitro* models)	Year	*In vitro* model	Main end points	Main outcome
Hosseini et al. (150)	2017	Isolated human primordial and primary follicles cultured to the preantral stage in a 3D alginate system	Follicle survival, follicle growth, transition to preantral stage	PRP promoted survival and development of isolated human primordial/primary follicles toward the preantral stage
Taghizabet et al. (19)	2022	Mouse preantral follicles cultured *in vitro*	Follicle growth, oocyte maturation, cleavage rate, apoptotic gene expression	Combination of conditioned medium and PRP improved follicle growth and cleavage rate; PRP alone was less consistently effective
Lange-Consiglio et al. (20)	2023	Bovine ovarian sections and granulosa-cell culture, including LPS-induced inflammatory stress	Granulosa-cell proliferation, apoptosis, inflammatory response, estradiol, AMH	PRP stimulated granulosa-cell proliferation and counteracted inflammatory processes *in vitro*
Subiran Adrados et al. (18)	2023	Cultured human preantral follicles	Follicle survival, follicle growth, culture performance	Human platelet lysate significantly improved survival and growth of cultured human preantral follicles compared with FBS, HSA, and umbilical-cord plasma
Subiran Adrados et al. (151)	2024	Murine preantral follicle culture	Follicle survival, follicle growth, oocyte maturation, estradiol secretion, Gdf9/Bmp15 expression	PRP-derived products did not improve murine follicle-culture outcomes compared with FBS; activated UCP increased Gdf9 and Bmp15 mRNA
El Bakly et al. (21)	2020	Ovarian tissue molecular analysis in a galactosemia-induced POI rat model; relevant mechanistic PRP-releasate study	miR-223, β1-integrin, p70S6K, MCL-1, follicle number, follicular maturation, FSH, estradiol, AMH, inhibin B	O-rPRP inhibited excessive follicular atresia, activated mTOR-related signaling, and reduced apoptosis
Subiran Adrados et al. (151)	2024	Human ovarian tissue xenotransplanted into immunodeficient mice; *ex vivo*/*in vivo* tissue model	Vascularization, follicle survival, VEGF/Vegf, BAX/BCL2, HMOX1, inflammatory markers	Platelet-derived products did not significantly improve vascularization or follicle survival in xenotransplanted human ovarian tissue

Human follicle-culture studies suggested a potential beneficial effect of platelet-derived products on early follicular development. Hosseini et al. showed that PRP promoted survival and development of isolated human primordial and primary follicles cultured toward the preantral stage in a three-dimensional alginate system (150). Similarly, Subiran Adrados et al. reported that human platelet lysate improved survival and growth of cultured human preantral follicles compared with fetal bovine serum, human serum albumin, and umbilical-cord plasma (18).

Animal and cellular models also suggested possible effects on granulosa-cell function and follicular survival. Taghizabet et al. found that the combination of conditioned medium and PRP improved follicle growth and cleavage rate in cultured mouse preantral follicles, although PRP alone was less consistently effective (19). Lange-Consiglio et al. showed that PRP stimulated granulosa-cell proliferation and counteracted inflammatory processes in bovine ovarian sections and granulosa-cell culture exposed to inflammatory stress (20). El Bakly et al. further reported that optimized PRP releasate inhibited excessive follicular atresia, activated mTOR-related signaling, and reduced apoptosis in a galactosemia-induced premature ovarian insufficiency model (21).

However, not all preclinical models showed benefit. Subiran Adrados et al. reported that PRP-derived products did not improve murine follicle-culture outcomes compared with fetal bovine serum, although activated umbilical-cord plasma increased Gdf9 and Bmp15 mRNA expression. Overall, the *in vitro* and cellular literature suggests that PRP-related products may support follicle survival, follicular growth, granulosa-cell proliferation, anti-inflammatory responses, anti-apoptotic signaling, and mTOR-related survival pathways. Nevertheless, the effects were not uniform across models. The available evidence indicates that PRP activity is likely context-dependent and influenced by the biological model, platelet-derived preparation, follicular stage, and endpoint assessed.

#### 3.1.3 Animal evidence

Animal studies provided preclinical evidence that PRP may exert protective or restorative effects on ovarian tissue in models of ovarian injury, premature ovarian insufficiency, diminished ovarian reserve, and PCOS-like ovarian dysfunction. Most studies used rat models, while one study used mice with chemotherapy-induced diminished ovarian reserve (Table 5).

**Table 5:** Animal experimental studies.

Study	Year	Animal model	Molecular mechanism	Main ovarian outcome	Mechanistic relevance
Pazoki et al. (152)	2015	Mice	Platelet lysate provides growth-factor-rich supplementation as an alternative to FBS for follicular culture.	Supported pre-antral follicle growth and development *in vitro*.	Indicates platelet-derived factors may enhance folliculogenesis and follicle survival in culture systems.
Pazoki et al. (153)	2016	Mice	Platelet lysate likely acts through platelet-derived growth factors and trophic mediators that support cytoplasmic/nuclear maturation, especially when granulosa-cell support is absent.	Improved maturation rate in denuded oocytes, with positive effects on fertilisation and embryo development.	Suggests platelet lysate can partly substitute for granulosa-cell-derived paracrine support during oocyte maturation.
Dehghani et al. (18)	2018	Rats	Ovarian stereology, follicle counts, ovarian structure, sex hormone-related functional assessment	PRP was reported to improve ovarian structure and function in cyclophosphamide-induced ovarian failure	Early preclinical evidence that PRP may support follicular preservation or recovery after chemotherapy-induced ovarian injury.
Vural et al. (21)	2019	Rats	Granulosa cells, oocytes, theca cells, ovarian function, VEGF-related regenerative context	PRP was evaluated as a regenerative intervention in POI, alongside VEGF/MSC-based approaches	Supports the concept that PRP may act through angiogenic and stromal-regenerative mechanisms in ovarian injury models.
Anvari et al. (23)	2019	Rats with PCOS	Ovarian antioxidant potential, hormonal interaction, folliculogenesis-related changes	PRP was reported to improve ovarian antioxidant potential and folliculogenesis in a PCOS model	Suggests PRP may affect ovarian dysfunction through antioxidant, endocrine, and folliculogenic modulation rather than only tissue repair.
Ozcan et al. (19)	2020	Rats	Ovarian function, follicular injury protection, chemotherapy-related ovarian damage endpoints	PRP showed a protective effect on ovarian function after cyclophosphamide exposure	Supports an ovarian-protective role of PRP in chemotherapy-associated gonadotoxicity.
Ahmadian et al. (22)	2020	Rats with POI	Angiogenesis-related markers, ovarian tissue function, ovulation rate	PRP promoted ovarian “rejuvenation” and restored ovulation rate in the rat POI model	Linking PRP response to angiogenesis modulation and restoration of ovulatory activity.
El bakly et al. (27)	2020	Rats	miR-223, β1-integrin, p70S6K, MCL-1, FSH, estradiol, AMH, inhibin B, follicle number/maturation	O-rPRP reduced excessive follicular atresia and improved follicular maturation	PRP releasate activated mTOR-related signaling and inhibited apoptosis through increased p70S6K and MCL-1 expression.
Budak et al. (20)	2022	Rats	miR-223, β1-integrin, p70S6K, MCL-1, FSH, estradiol, AMH, inhibin B, follicle number/maturation	PRP showed protective effects on IVF-related outcomes in rats with cyclophosphamide-induced ovarian failure	Connects ovarian PRP exposure with functional reproductive outcomes, not only histological recovery.
Cozzolino et al. (25)	2024	Mice	Oocyte quality, early embryo development, ovarian reserve impairment after chemotherapy	PRP improved oocyte quality and early embryo development in chemotherapy-induced DOR mouse models	Linking PRP not only to follicle number but also to oocyte competence and embryo-development potential.
Alaee et al. (154)	2024	Mice	PRP supplementation in IVM medium appeared to reduce apoptosis-related signaling and increase anti-apoptotic Bcl2l1 expression.	Increased oocyte maturation, fertilisation, and subsequent embryo development.	Supports a direct pro-maturation and pro-survival effect of PRP on immature oocytes during *in vitro* maturation.
Zhao et al. (24)	2025	Rats	Ddx4, PCNA, BMP4, CD34, BCL2, Caspase-3, AMH, FSHR, oxidative stress markers, mitochondrial membrane potential, DNA repair proteins, proteomics/metabolomics	PRP improved sex hormone levels and ovarian molecular markers; reduced oxidative stress, mitochondrial damage, DNA damage, and inflammation	PRP action through germ-cell markers, granulosa-cell survival, angiogenesis, oxidative-stress reduction, DNA repair, and inflammatory modulation.
Fotoohi-Ardakani et al. (26)	2025	Rats with POI	Ovarian recovery parameters; dose-response effect	PRP effects differed according to concentration	PRP heterogeneity and the need for standardized dose/concentration protocols.
Weng et al. (155)	2025	Newborn mouse ovary; human ovarian cortex *in vitro*	hPRP activates PI3K/Akt → p-FOXO3a, causing FOXO3a nuclear export	More growing follicles; reduced primordial follicle pool proportion	PRP may activate dormant follicles via oocyte PI3K/Akt signaling

In chemotherapy-induced ovarian injury models, PRP was generally associated with improved ovarian structure and function. Dehghani et al. reported improved ovarian morphology, follicle counts, and sex hormone-related parameters after PRP administration in cyclophosphamide-induced ovarian failure (22). Ozcan et al. similarly showed a protective effect of PRP on ovarian function after cyclophosphamide exposure (23). Budak et al. extended these findings by showing that PRP had protective effects on IVF-related outcomes in rats with cyclophosphamide-induced ovarian failure, suggesting that PRP may influence functional reproductive outcomes as well as histological recovery (24).

Several studies suggested that PRP may act through angiogenic, stromal-regenerative, antioxidant, and anti-inflammatory mechanisms. Vural et al. evaluated PRP as a regenerative intervention in premature ovarian insufficiency alongside VEGF- and mesenchymal stem-cell-based approaches, supporting the relevance of angiogenic and stromal repair pathways (25). Ahmadian et al. reported that PRP promoted ovarian recovery and restored ovulation rate in a rat POI model, with mechanistic relevance to angiogenesis-related markers and ovarian tissue function (26). Anvari et al. showed that PRP improved antioxidant potential and folliculogenesis-related changes in a rat PCOS model, indicating that PRP effects may extend beyond chemotherapy-induced injury (156).

More detailed molecular studies supported involvement of survival and anti-apoptotic pathways. El Bakly et al. reported that optimized PRP releasate reduced excessive follicular atresia and improved follicular maturation, with changes involving miR-223, β1-integrin, p70S6K, MCL-1, AMH, inhibin B, FSH, and estradiol (21). Zhao et al. further supported a broad molecular effect of PRP in a cyclophosphamide-induced POI rat model, showing improved sex hormone levels and ovarian molecular markers, together with reduced oxidative stress, mitochondrial damage, DNA damage, and inflammation (108).

Mouse data also suggested that PRP may affect oocyte competence. Cozzolino/Dai et al. reported improved oocyte quality and early embryo development after PRP treatment in chemotherapy-induced diminished ovarian reserve models (157). Finally, Fotoohi-Ardakani et al. showed that PRP effects differed according to concentration in a rat POI model, highlighting the importance of PRP preparation, dose, and standardization (158).

Pazoki et al. investigated platelet lysate as a growth-factor-rich alternative to fetal bovine serum for murine follicular culture and reported that it supported pre-antral follicle growth and development *in vitro*. These findings suggest that platelet-derived soluble factors may contribute to folliculogenesis and follicle survival within culture systems (152). In a related murine study, Pazoki et al. further examined the role of platelet lysate during oocyte maturation, particularly in denuded oocytes lacking granulosa-cell support. Platelet lysate improved oocyte maturation rates and was associated with positive effects on fertilisation and subsequent embryo development (153).

From a different perspective, Subiran Adrados et al. investigated the potential role of platelet-rich plasma-derived products in improving graft revascularization and follicular survival following xenotransplantation of human ovarian tissue. Although vascularization, assessed by CD31-positive area, increased between day 3 and day 6 after transplantation, this improvement was not significantly greater in the treated groups compared with controls. In addition, VEGF expression decreased over time in both treated and control grafts, while the BAX/BCL-2 ratio was significantly increased in the human platelet lysate group, suggesting a possible shift toward apoptosis rather than enhanced graft protection. Follicle density after 4 weeks was also not significantly improved by human platelet lysate or umbilical cord plasma compared with controls. Overall, these findings suggest that, despite the proposed angiogenic and regenerative potential of platelet-rich plasma, the specific PRP-derived preparations and protocol used in this xenotransplantation model did not improve early graft vascularization or follicular survival (150).

Interestingly, there are also data indicating that PRP supplementation during mouse oocyte *in vitro* maturation improved maturation, fertilisation, and subsequent embryo development. This effect was associated with reduced apoptosis-related signalling and increased anti-apoptotic Bcl2l1 expression, supporting a direct pro-maturation and pro-survival role of PRP in immature oocytes (154).

Finally, Weng et al. (155) demonstrated that human platelet-rich plasma promoted primordial follicle activation in newborn mouse ovaries and cultured human ovarian cortex by activating the PI3K/Akt–FOXO3a pathway, resulting in an increased proportion of growing follicles.

### 3.2 Computational analysis

**Figure 2:**
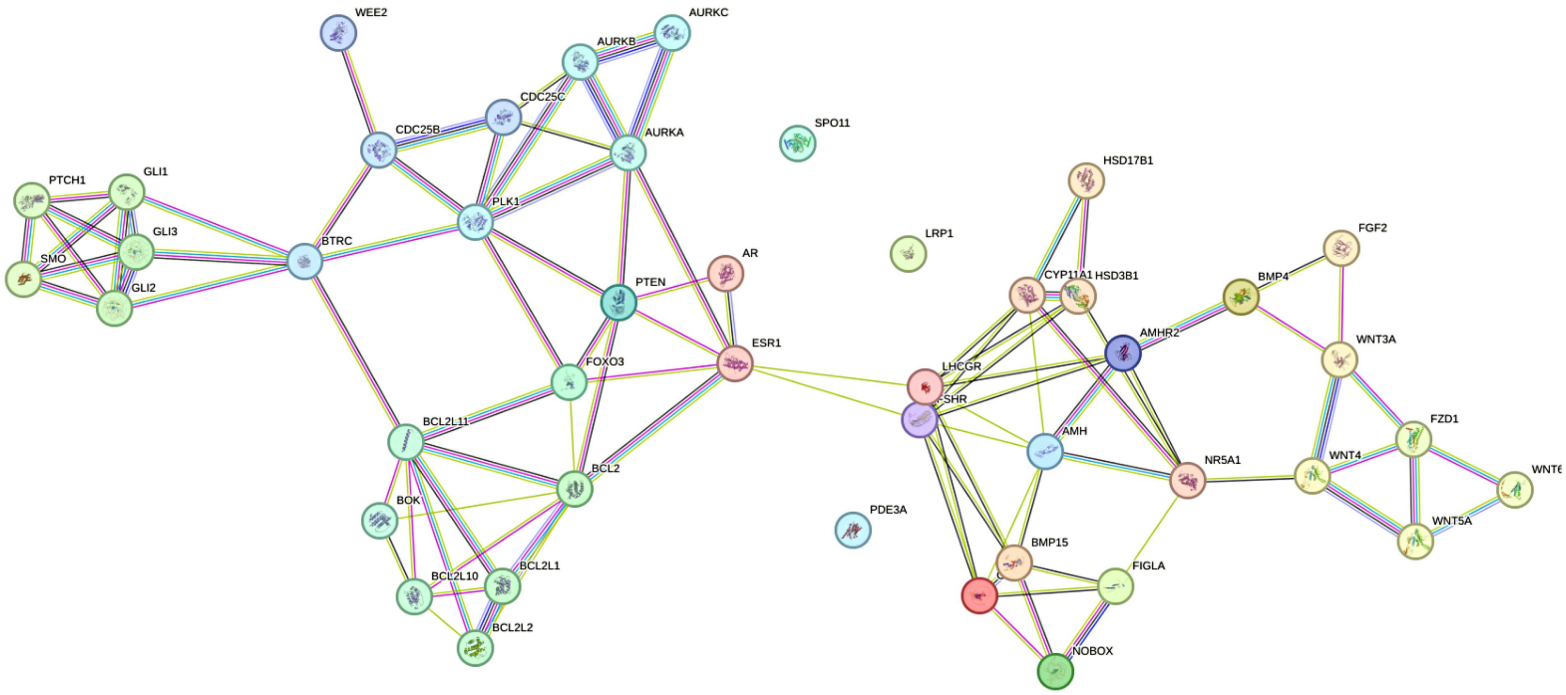
Protein–protein interaction network and gene mapping for folliculogenesis-associated genes. The network was generated using the STRING database for Homo sapiens protein–protein interactions. Only the predefined folliculogenesis-associated gene set was included, without addition of first-shell or second-shell interactors. A high-confidence interaction score threshold of ≥0.700 was applied. Nodes represent proteins encoded by folliculogenesis-associated genes identified from the literature review. Node colours indicate functional gene categories within folliculogenesis, including oocyte-specific transcriptional regulators, oocyte-derived paracrine mediators, primordial follicle activation and survival factors, granulosa-cell differentiation markers, gonadotropin and hormone receptors, steroidogenic enzymes, angiogenic factors, and intracellular signaling mediators. Edges represent protein–protein associations supported by STRING evidence; edge colours correspond to the type of supporting evidence, and thicker lines indicate higher confidence interaction scores. Disconnected nodes were retained in the analysis to preserve the complete predefined gene set.

**Figure 3:**
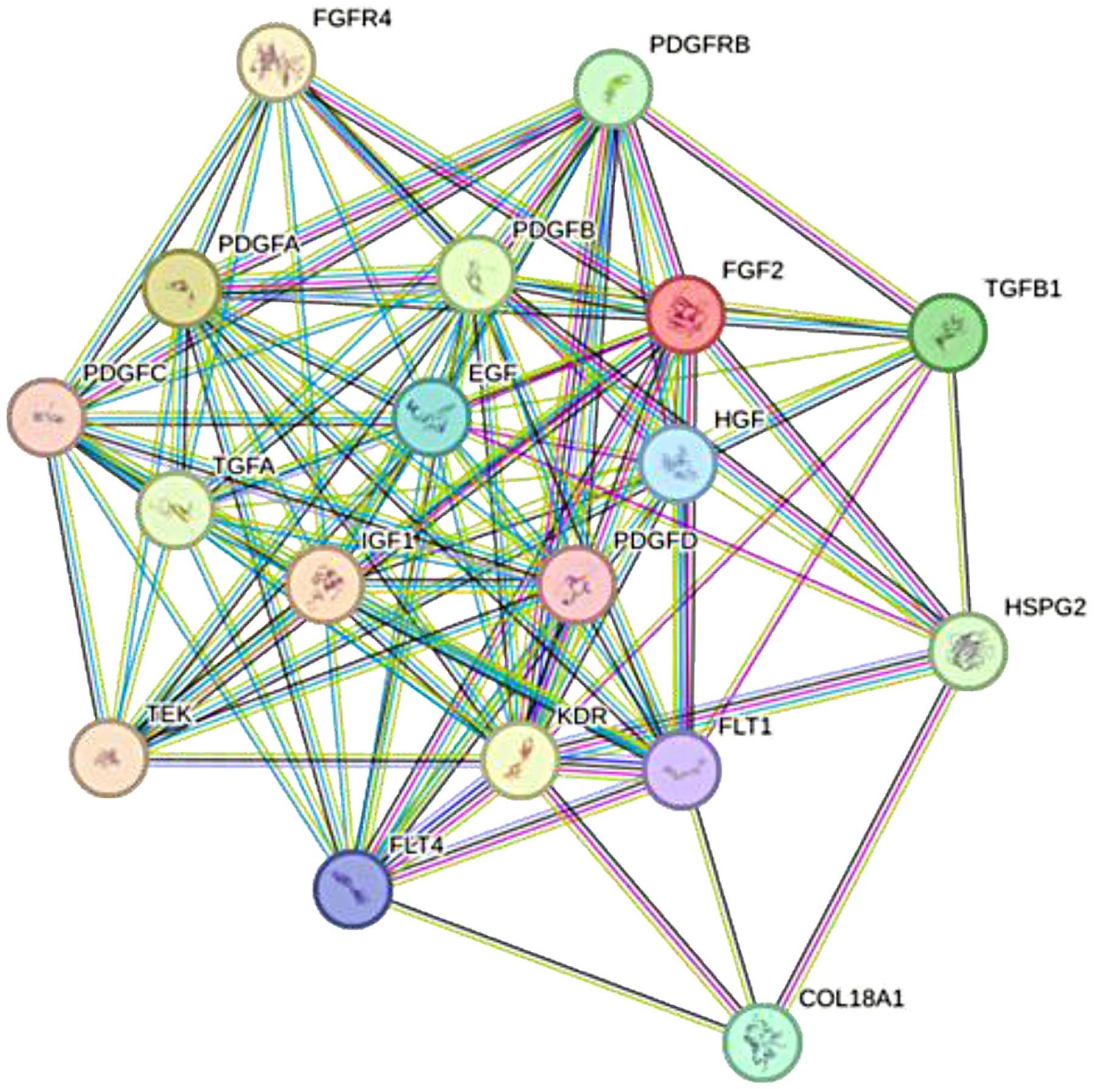
Protein–protein interaction network of PRP-associated mediators. The network was generated using the STRING database for Homo sapiens protein–protein interactions. The analysis included the predefined platelet-rich plasma (PRP)-associated gene set, comprising PRP-related growth factors, cytokines, receptors, angiogenic mediators, extracellular-matrix remodeling molecules, inflammatory mediators, and intracellular signaling components identified from the literature review. No first-shell or second-shell interactors were added. A high-confidence STRING interaction score threshold of ≥0.700 was applied. Nodes represent proteins encoded by PRP-associated genes. Edges represent protein–protein associations supported by STRING evidence. Edge colours indicate the source of interaction evidence, including experimentally validated interactions, curated database evidence, co-expression, and other STRING evidence channels where retained in the network output. Thicker edges indicate higher confidence interaction scores. Disconnected nodes were retained to preserve the complete predefined PRP-associated gene set.

The investigation of possible molecular mechanisms through which PRP may affect follicle function was performed through an integrated network and functional bioinformatics analysis. Initially, two independent gene sets were created, the first consisting of genes involved in folliculogenesis (Table 2) and the second of growth factors and related receptors described in PRP biology (Table 1). For each set, protein–protein interactions analysis was performed using the STRING database (Figures 2, 3), followed by functional enrichment of biological processes and signaling pathways through the KEGG database.

When comparing the nodes of the two networks, only limited gene overlap was revealed. Comparison of the two gene sets showed limited direct overlap, with fibroblast growth factor 2 (FGF2) identified as the only shared gene. FGF2 was present in both the PRP-associated and folliculogenesis-associated gene sets and is annotated in the literature in relation to angiogenesis and granulosa-cell proliferation (108, 156). Although there is only limited direct gene overlapping between the two networks, when comparing the pathway content of the two networks by performing KEGG and Reactome pathway enrichment, we observed statistically significant overlap of pathways (*p*-value = 0.05, FDR < 0.05) related to receptor tyrosine kinases (RTKs) such as PI3K-Akt signaling, MAPK signaling and FoxO-mediated survival signaling.

**Figure 4:**
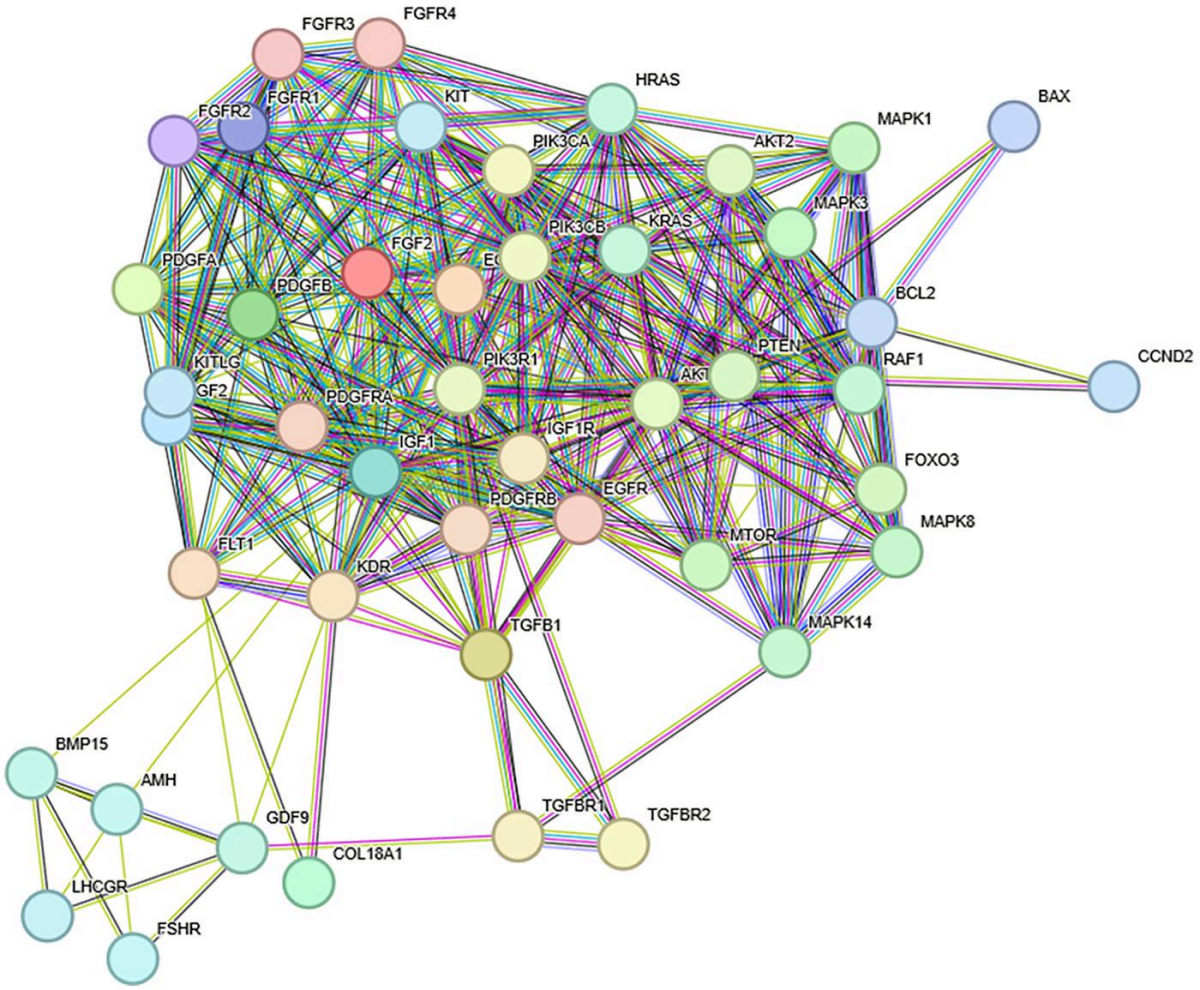
Integrated protein–protein interaction network of PRP-associated mediators and folliculogenesis-associated genes. The network was generated using the STRING database for Homo sapiens protein–protein interactions and includes the combined predefined gene sets derived from the systematic review: the PRP-associated gene set and the folliculogenesis-associated gene set. No first-shell or second-shell interactors were added. A high-confidence STRING interaction score threshold of ≥0.700 was applied. Nodes represent proteins encoded by genes from the two predefined datasets. Node colours distinguish gene-set membership: one colour indicates PRP-associated genes, a second colour indicates folliculogenesis-associated genes, and a third colour indicates genes shared by both datasets where applicable. This colour scheme allows visual assessment of the degree of direct overlap and indirect connectivity between the two gene sets within the integrated network. Edges represent protein–protein associations supported by STRING evidence; edge colours indicate the source of supporting evidence, and thicker lines indicate higher confidence interaction scores. Disconnected nodes were retained in order to preserve the complete combined predefined gene set.

To further evaluate direct mechanistic integration, a combined gene set including all PRP-associated and folliculogenesis-associated genes was subjected to high-confidence STRING PPI analysis score ≥ 0.700; no additional interactors added) (Figure 4). The resulting network demonstrated a densely interconnected receptor tyrosine kinase–centered architecture. The endogenous transcriptional regulatory mechanisms controlling folliculogenesis include the SOHLH-FIGLA-NOBOX axis and the oocyte derived factors GDF9 and BMP15 whereas the activation of primordial follicles into activation is dependent upon PI3K-AKT-FOXO signaling, with major regulators being FOXO3 and PTEN.

In this network, FGF2 showed direct interaction with its canonical receptors FGFR1–FGFR4. Downstream connectivity extended to PIK3CA, PIK3R1, AKT1, AKT2, MTOR, PTEN, and components of the RAS–RAF–MAPK cascade, including KRAS, RAF1, MAPK1, and MAPK3. Together, these interactions define two coherent signaling routes, one linking FGF2–FGFR activation to PI3K/AKT signaling and the other linking FGF2–FGFR activation to RAS/RAF/MAPK signaling. These routes provide a biologically plausible framework through which PRP-associated growth-factor input may converge on follicular survival, proliferation, and microenvironmental support pathways.

Functional enrichment of the network combining both datasets confirmed that PI3K–Akt signaling (KEGG hsa04151) and MAPK signaling (KEGG hsa04010), and Reactome PI3K Cascade Pathways were significantly enriched (FDR < 0.05). These results suggested a strong consensus for functional convergence at the most downstream canonical survival and proliferation pathways.

Regulation of folliculogenesis is mediated by endogenous transcriptional networks involving the SOHLH–FIGLA–NOBOX axis (168) and oocyte-derived paracrine mediators, including GDF9 and BMP15 (169). Primordial follicle activation is critically regulated by intracellular signaling pathways, particularly the PI3K–AKT–FOXO pathway and its major regulators, FOXO3 and PTEN (167). As the present network analysis does not support direct substitution of this intrinsic transcriptional program by PRP-derived factors, results indicate convergence at the level of shared receptor tyrosine kinase-mediated survival signaling pathways.

Collectively, the above-mentioned findings support a model in which PRP influences Follicular Biology primarily through upstream growth factor signaling enhancement and amplification of endogenous PI3K-AKT-MAPK survival pathways within the ovarian microenvironment.

## 4 Discussion

Based on the available clinical, *in vitro*, and animal literature, PRP has been proposed to influence ovarian function primarily through microenvironmental mechanisms, including angiogenesis, stromal remodeling, inflammatory modulation, and granulosa-cell support. Available evidence instead supports the interpretation that PRP may act primarily as a modulator of the ovarian microenvironment, influencing stromal, vascular, inflammatory, and granulosa-cell support pathways around existing follicles. In the ovarian context, these mechanisms support the hypothesis that PRP provides an upstream paracrine stimulus capable of modifying the stromal, vascular, and granulosa-cell environment in which follicular development occurs. Angiogenesis is one of the most consistently proposed mechanisms, with VEGF-related vascular remodeling potentially improving oxygen delivery, stromal support, and follicular survival, particularly in diminished ovarian reserve and premature ovarian insufficiency models (13, 159). In parallel, PI3K–AKT and MAPK/ERK signaling are established pathways involved in ovarian follicular development, including follicle activation, granulosa-cell survival and proliferation, steroidogenic activity, cumulus–oocyte complex function, oocyte maturation, ovulation, and ovarian-cell survival (41, 108, 160, 161).

Additional mechanisms proposed in the literature include mTOR-related growth and survival signaling, anti-apoptotic effects, inflammatory modulation, oxidative-stress reduction, mitochondrial support, extracellular-matrix remodeling, and extracellular vesicle-mediated paracrine communication (12, 13, 162, 163). mTOR signaling has been linked to follicular activation, cellular metabolism, and resistance to atresia, while PRP-associated anti-apoptotic effects may preserve granulosa-cell viability and reduce follicular loss in ovarian injury models (13, 162). Reviews also suggest that PRP may reduce inflammatory and oxidative stress and support mitochondrial function, mechanisms that are relevant to oocyte quality, granulosa-cell function, and ovarian aging (162, 163). Taken together, the available review literature supports a model in which PRP may enhance ovarian function by improving the permissive microenvironment for follicular survival and growth. Only one recent publication reported PRP-induced primordial follicle activation through the PI3K/Akt–FOXO3a pathway; however, it remains the only study of this type, and its findings should therefore be interpreted with caution (155). Accordingly, PRP should be interpreted as a potential microenvironmental modulator that may support follicular survival, granulosa-cell function, stromal repair, vascular remodeling, and ovarian responsiveness. This framing is consistent with the pathway-level convergence observed in the computational analysis and avoids attributing mechanisms to PRP that were not directly tested in the included studies. Any potential effect of PRP is therefore more appropriately understood as support of residual follicles and their surrounding ovarian microenvironment, rather than induction of *de novo* folliculogenesis or replacement of the intrinsic oocyte-specific developmental program. Therefore, PRP should be interpreted as a potential microenvironmental modulator that may support follicular survival, granulosa-cell function, stromal repair, and ovarian responsiveness, rather than as a direct inducer of *de novo* folliculogenesis.

Importantly, the clinical evidence should be interpreted according to methodological quality. Although case reports and uncontrolled observational studies have reported improvements in AMH, AFC, follicular response, oocyte yield, embryo development, and occasional pregnancies after intraovarian PRP, these study designs cannot reliably distinguish a true treatment effect from selection bias, spontaneous biological variability, regression to the mean, or concurrent changes in stimulation protocols. The most methodologically rigorous evidence is therefore more informative. In this context, the PROVA randomized placebo-controlled trial is particularly important because it did not show significant improvement in mature oocyte yield, blastocyst yield or euploidy, AMH, or AFC after intraovarian PR P (136). This null result substantially limits the strength of clinical claims and supports a cautious interpretation: while PRP remains biologically plausible and mechanistically interesting, its clinical efficacy for improving ovarian response or IVF outcomes has not yet been demonstrated in robust randomized evidence. Additionally, some of the observed effects following intraovarian PRP injection may partly reflect the biological response to ovarian tissue injury, since mechanical disruption of ovarian tissue has been shown to influence follicle activation pathways, including Hippo/actin-related signaling, and to induce inflammatory and tissue-remodeling responses. Therefore, in uncontrolled observational studies and case reports, the intraovarian injection procedure itself should be considered a potential confounding factor when interpreting PRP-related effects (164, 165).

A second major interpretive issue is the heterogeneity of PRP itself. PRP is not a standardized product, and differences in platelet concentration, leukocyte content, activation method, centrifugation protocol, injected volume, and growth-factor/cytokine composition may alter its biological activity. Therefore, the PRP-associated gene set used in the present analysis should be viewed as a generalized mediator framework rather than the molecular profile of a single PRP formulation. The observed pathway convergence supports biological plausibility, but it should not be assumed that all PRP preparations activate the same pathways or produce equivalent ovarian effects.

Our review and computational analysis further refine this interpretation by distinguishing direct molecular overlap from pathway-level convergence. Direct gene overlap between the PRP-associated mediator set and the folliculogenesis-associated gene set was limited, with FGF2 identified as the only shared molecule. This finding is mechanistically important because FGF2 is both a PRP-associated growth factor and an established ovarian regulator involved in angiogenesis, granulosa-cell proliferation, and early follicular recruitment. In the integrated STRING network, FGF2 showed direct connectivity with FGFR1–FGFR4, and downstream links extended to PIK3CA, PIK3R1, AKT1, AKT2, MTOR, PTEN, and the RAS–RAF–MAPK cascade, including KRAS, RAF1, MAPK1, and MAPK3. These findings suggest a coherent signaling architecture through which PRP-derived growth factor input may be transmitted to intracellular pathways involved in follicular survival, proliferation, and microenvironmental support.

The present findings should therefore be interpreted in the context of established ovarian biology: the human ovarian reserve consists of a finite pool of non-growing follicles established before birth, which declines progressively with age. The present findings should therefore be interpreted in the context of the existing ovarian follicular pool and its surrounding microenvironment (166). The computational findings indicate convergence at the level of receptor tyrosine kinase-mediated survival pathways, particularly PI3K–AKT, MAPK, mTOR, and FOXO/PTEN-related signaling (41, 155, 160, 161). This supports a model in which PRP may enhance upstream growth-factor signaling and amplify endogenous survival and proliferation pathways within the ovarian microenvironment (12, 13, 162, 163).

Instead, the computational findings indicate convergence at the level of shared receptor tyrosine kinase-mediated survival pathways, particularly PI3K–AKT, MAPK, mTOR, and FOXO/PTEN-related signaling. This supports a model in which PRP acts primarily by enhancing upstream growth-factor signaling and amplifying endogenous survival and proliferation pathways within the ovarian microenvironment, rather than initiating *de novo* folliculogenesis. In this context, PRP may improve follicular competence indirectly by supporting granulosa-cell survival, angiogenesis, anti-apoptotic signaling, stromal repair, and oxidative-stress/inflammatory modulation, while the core developmental program remains dependent on endogenous oocyte–granulosa regulatory mechanisms.

Overall, the present findings should be interpreted within a mechanistic framework in which PRP acts as a biological amplifier of permissive ovarian signaling rather than as an autonomous inducer of folliculogenesis. The limited gene-level overlap between PRP-associated mediators and established folliculogenesis regulators suggests that PRP is unlikely to substitute the intrinsic oocyte-specific developmental machinery. Instead, the observed pathway-level convergence supports the hypothesis that PRP may enhance the ovarian microenvironment through growth-factor-driven activation of receptor tyrosine kinase signaling, angiogenic remodeling, anti-apoptotic responses, stromal repair, and amplification of PI3K–AKT, MAPK/ERK, and mTOR-related survival pathways. This interpretation may also explain the heterogeneity of clinical outcomes reported in the literature: PRP may be more effective when residual follicles and functional granulosa–stromal signaling networks are still present, whereas its effect may be limited in cases of advanced follicular depletion.

In conclusion, this review and computational analysis provide a systems-level rationale for the biological plausibility of PRP in ovarian regenerative medicine. The available evidence supports a model in which PRP may influence follicular survival, granulosa-cell function, vascular support, and ovarian tissue responsiveness through modulation of the ovarian microenvironment. Future studies should move beyond descriptive clinical outcomes and incorporate standardized PRP characterization, defined patient selection, controlled study designs, and molecular validation of the predicted pathways in ovarian tissue or follicular-cell models. Such studies will be essential to determine whether the pathway convergence identified here translates into reproducible biological and clinical benefit.

### 4.1 Limitations

The findings of this study should be interpreted in light of the heterogeneity of the evidence base. The review included clinical case reports, observational studies, randomized trials, *in vitro* studies, and animal models, which do not provide the same level of evidence. Case reports and uncontrolled studies are particularly vulnerable to selection bias, spontaneous cycle-to-cycle variability, regression to the mean, and placebo effects. Although greater interpretive weight was assigned to randomized controlled evidence when evaluating clinical efficacy, the heterogeneity of study designs prevented quantitative weighting of all included studies by methodological quality.

Another limitation is that this review was not prospectively registered in PROSPERO or another systematic-review registry before data collection and study screening. This limits transparency regarding the *a priori* specification of the review protocol and means that protocol-level deviations cannot be fully excluded.

A supplementary clinical-trial registry search was performed to identify ongoing, unpublished, or registry-only studies evaluating intraovarian or ovarian PRP. However, registry records without posted results or extractable outcome data could not be incorporated into the main evidence synthesis and were reported separately. Therefore, the possibility of unpublished negative studies, incomplete outcome reporting, or selective outcome availability remains a limitation.

Risk of bias was assessed according to study design and used to guide interpretation of the evidence. However, because the included evidence consisted of randomized trials, observational studies, case reports, *in vitro* studies, and animal experiments, a single uniform risk-of-bias tool could not be applied across all evidence categories. This limits direct comparability between study types and prevents a fully standardized methodological-quality assessment across the entire evidence base.

A further limitation concerns the heterogeneity of PRP preparation. PRP is not a standardized biological product, and studies differ in platelet concentration, leukocyte content, activation method, centrifugation protocol, injection volume, growth-factor and cytokine composition, extracellular-vesicle content, and release kinetics. Therefore, studies using the term “PRP” may in fact be evaluating biologically different preparations. This variability limits the interpretation of pooled molecular findings, because pathway-level convergence identified in the computational analysis cannot be assumed to apply equally to all PRP formulations.

Another potential limitation of this review is the variability in terminology used to describe platelet-rich plasma and related platelet-derived preparations across the literature. Although the search strategy included established terms such as “platelet-rich plasma” and “PRP,” and related nomenclature such as “platelet lysate,” “platelet concentrate,” “autologous conditioned plasma,” and “platelet-derived products” was also considered where relevant, some studies may have used alternative or non-standard terminology. To minimize this risk, abstracts and full publications were reviewed to assess whether the product described was relevant to platelet-rich plasma or related platelet-derived preparations. Nevertheless, it remains possible that relevant studies using different nomenclature were not identified or included.

Finally, the computational component of this study remains hypothesis-generating. STRING-based protein–protein interaction modeling and pathway enrichment analysis can identify molecular connectivity and pathway-level convergence, but they do not demonstrate pathway activation in ovarian tissue. These analyses cannot account fully for tissue-specific protein expression, cell-type-specific signaling, temporal pathway dynamics, ligand concentration, post-translational modifications, or the bioavailability of PRP-derived mediators after intraovarian administration. Therefore, the computational findings should be interpreted as a mechanistic framework requiring experimental validation rather than as direct evidence of biological activity in human ovarian tissue.

### 4.2 Implications

The present findings have direct implications for future research design. Future studies should prioritize adequately powered randomized, placebo- or sham-controlled trials, because changes in ovarian reserve markers after PRP may reflect spontaneous variability, regression to the mean, or selection bias rather than a true biological treatment effect. This is particularly important given the discrepancy between encouraging observational studies and the null findings reported in the most rigorous randomized evidence. Patient selection should also be more precise, and poor ovarian response, diminished ovarian reserve, premature ovarian insufficiency, perimenopause, and menopause should not be treated as interchangeable indications.

PRP preparation should be standardized and fully reported, including baseline platelet count, final platelet concentration, leukocyte content, activation method, centrifugation protocol, injected volume, number and timing of injections, and, where possible, growth-factor and cytokine profiling. Future studies should also include clinically meaningful reproductive outcomes, such as mature oocyte yield, blastocyst formation, euploidy, clinical pregnancy, miscarriage, and live birth, rather than relying only on AMH or AFC. Finally, mechanistic endpoints should be incorporated to validate whether PRP activates the predicted pathways, including PI3K–AKT, MAPK/ERK, mTOR, FOXO/PTEN, VEGF, FGF/FGFR, apoptosis, oxidative-stress, inflammatory, mitochondrial, vascular, stromal, and granulosa-cell markers.

## Data Availability

The original contributions presented in the study are included in the article/Supplementary Material, further inquiries can be directed to the corresponding authors.
