# Supplementary material for: Platelet-rich plasma and folliculogenesis: a systematic mechanistic review and computational pathway analysis
Source: Front Reprod Health. 2026 Jul 30;8:1883067. doi: 10.3389/frph.2026.1883067 (PMC13468918; doi:10.3389/frph.2026.1883067)
Supplement: Supplementary file 1 [file Table1.docx]

**Table 1**

| Trial ID | Relevance |
| --- | --- |
| NCT03542708 | **Autologous PRP injections in women with primary ovarian insufficiency; one randomly selected ovary treated; no results posted in the registry record.** |
| NCT04275700 | **PRP in women with diminished ovarian reserve.** |
| NCT05279560 | **Ovarian PRP vs saline placebo for follicular activation in low ovarian reserve/expected poor ovarian response before IVF/ICSI** |
| NCT05790655 | **Prospective double-blind randomized placebo-controlled ovarian PRP study in diminished ovarian reserve.** |
| NCT04163640 | **Prospective randomized controlled trial of intraovarian PRP in women with POI and POR declining donor oocytes.** |
| NCT04381299 | **Autologous PRP in women with POI undergoing IVF/ICSI.** |
| NCT04149028 | **Platelet-rich plasma injection into the ovary of patients with POI.** |
| NCT03178695 | **“Inovium Ovarian Rejuvenation Trials”; PRP in diminished ovarian reserve/poor follicular recruitment.** |
| NCT05385848 | **Pilot study of autologous PRP infusion for DOR/POI and IVF outcomes.** |
| NCT05105724 | **Autologous PRP to improve responsiveness and embryo quality in poor ovarian response** |
| NCT05601193 | **Ovarian PRP injection in women with POR; listed as unknown status, last known recruiting.** |
| NCT06773572 | **Autologous exosomes versus PRP for ovarian biostimulation/bioregeneration in low ovarian reserve.** |
| NCT06663930 | **New approach for ovarian PRP injection in poor responders.** |
| NCT07282314 | **Prospective RCT of autologous PRP local injection for POR/POI and IVF-ET outcomes** |
| 2020-000247-32 / MAXINOVO | **Directly relevant Spanish trial: activation of primordial follicles in poor ovarian reserve using growth factors; listed as ongoing, with no results available.** |
