# Supplementary material for: Platelet-rich plasma and folliculogenesis: a systematic mechanistic review and computational pathway analysis
Source: Front Reprod Health. 2026 Jul 30;8:1883067. doi: 10.3389/frph.2026.1883067 (PMC13468918; doi:10.3389/frph.2026.1883067)
Supplement: Supplementary file 2 [file Table2.docx]

**Table 2**

| Human gene symbol | Original reported gene/protein | Species/model | Evidence source | Reason for inclusion |
| --- | --- | --- | --- | --- |
| GDF9 | Gdf9 | Mouse follicle/oocyte model | Animal/in vitro folliculogenesis literature | Oocyte-derived regulator of follicular development |
| BMP15 | Bmp15 | Mouse follicle/oocyte model | Animal/in vitro folliculogenesis literature | Oocyte-derived paracrine regulator of follicular development |
| BCL2L1 | Bcl2l1 | Mouse oocyte/PRP maturation model | In vitro PRP/oocyte maturation study | Anti-apoptotic mediator linked to oocyte survival |
| BCL2 | Bcl2/BCL-2 | Animal ovarian injury/PRP models | Animal PRP/ovarian injury literature | Anti-apoptotic survival mediator |
| CASP3 | Caspase-3/Casp3 | Rat ovarian injury/PRP models | Animal PRP/ovarian injury literature | Apoptosis marker |
| MCL1 | Mcl1/MCL-1 | Rat PRP releasate/POI model | Animal PRP mechanistic literature | Anti-apoptotic mediator |
| ITGB1 | β1-integrin/Itgb1 | Rat PRP releasate/POI model | Animal PRP mechanistic literature | Cell adhesion and survival signaling |
| RPS6KB1 | p70S6K | Rat PRP releasate/POI model | Animal PRP/mTOR-related literature | mTOR downstream signaling mediator |
| DDX4 | Ddx4/VASA | Rat POI/PRP model | Animal ovarian reserve/PRP literature | Germ-cell/oocyte marker |
| PCNA | Pcna/PCNA | Rat POI/PRP model | Animal PRP/ovarian repair literature | Cell proliferation marker |
| BMP4 | Bmp4 | Animal folliculogenesis/PRP models | Animal follicular development literature | Follicular growth and ovarian signaling regulator |
| CD34 | Cd34/CD34 | Rat ovarian angiogenesis/PRP model | Animal PRP/ovarian vascular marker literature | Angiogenesis/endothelial marker |
| VEGFA | Vegfa/VEGF | Animal/in vitro ovarian tissue models | Animal/in vitro PRP or folliculogenesis literature | Angiogenic mediator |
| FOXO3 | Foxo3 | Mouse ovarian reserve/follicle dormancy models | Animal folliculogenesis literature | Primordial follicle dormancy/survival regulator |
| ESR1 | Esr1 | Animal ovarian function models | Animal folliculogenesis/endocrine signaling literature | Estrogen receptor signaling |
| ESR2 | Esr2 | Animal ovarian function models | Animal folliculogenesis/endocrine signaling literature | Granulosa-cell estrogen receptor signaling |
| AR | Ar | Animal/in vitro follicle activation models | Animal/in vitro folliculogenesis literature | Androgen receptor signaling in follicular development |
